# Simulation dataset of annual crop growth, GHG emission and SOC stock dynamics under current and projected climate conditions for major crops with current and reduced fertiliser inputs in Southwest, England

**DOI:** 10.1016/j.dib.2024.110574

**Published:** 2024-06-07

**Authors:** Yusheng Zhang, Lianhai Wu, Asma Jebari, Adrian L. Collins

**Affiliations:** Net Zero and Resilient Farming, Rothamsted Research, North Wyke, Okehampton, Devon EX20 2SB, UK

**Keywords:** Scenario, Net zero, Integrated modelling, Ecosystem services

## Abstract

For mitigating the unintended environmental impacts associated with intensive farming across the world, it is crucial to understand the complex impacts of potential reductions in fertiliser use on multiple ecosystem services, including crop production, GHG emissions and changes in soil organic carbon (SOC) stocks. Using site specific spatial data and information, a novel integrated modelling approach using established agroecosystem models (SPACSYS and RothC) was implemented to evaluate the impacts of various fertiliser reductions (10 %, 30 % and 50 %) under current / baseline and projected (RCP2.6, RCP4.5 and RCP8.5) climate scenarios in a study catchment in southwest England. 48 unique combinations of soil types, climate conditions and fertiliser inputs were evaluated for five major arable crops (winter wheat, maize, winter barley, spring barley, winter oilseed rape) plus ryegrass. Modelled annual estimates of crop yields and biomass, emissions of gases with warming potentials (nitrous oxide, methane, carbon) and SOC stocks in the topsoil (0–30 cm) were tabulated for all combinations considered. These simulated data series could be further analysed to evaluate inter-annual variations and their implications for climate resilience and combined with additional data to quantify nutrient use efficiency and undertake cost- benefit analysis, and to contribute to inter-regional comparisons of fertiliser management at broad scale.

Specifications Table

**Table:** 

Subject	Mathematic modelling
Specific subject area	Simulation of crop responses to changing climate conditions and reduced fertiliser rates
Data format	Raw, Analysed, Aggregated
Type of data	Table
Data collection	Data were generated mainly using two established agroecosystem models: SPACSYS [2,3] and RothC [4]. SPACSYS (version 6) was run in a SQL server where all inputs (parameters and data) and outputs were stored. A selection of outputs was extracted from the server to Excel worksheets using a customised routine based on Excel VBA for further processing. Simulated daily gas emissions, including nitrous oxide, methane and carbon dioxide from plants and soil throughout the year were summed to calculate annual emission intensities. Daily development of leaves, stems, grains and of the root system was simulated continuously throughout the growing season for the modelling years. Annual crop yields and biomass for different parts of the crop at a physiological mature stage, i.e., harvest date, were estimated. The estimated annual biomass productions were then used to characterise annual fresh SOC input to the soil for the RothC model to update SOC stocks in the topsoil layer. RothC was implemented and operated in an Excel environment (version Microsoft365). Only year-end SOC stocks were reported without corresponding monthly dynamics.
Data source location	Institution: Rothamsted Research City/Town/Region: Devon County Country: England
Data accessibility	Repository name: Zenodo Data identification number: DOI: 10.5281/zenodo.10700084 Direct URL to data: https://doi.org/10.5281/zenodo.10700084
Related research article	Zhang, Y., Wu, L., Jebari, A. and Collins, A. L. 2024. Impacts of reduced synthetic fertiliser use under current and future climates: Exploration using integrated agroecosystem modelling in the upper River Taw observatory, UK. Journal of Environmental Management. 351, 119,732. https://doi.org/10.1016/j.jenvman.2023.11973

## 1 Value of the Data

• Interactions between climate change, plant responses and fertilizer management, especially application rates, significantly affect the provision of multiple ecosystem services. This complex relationship tends to be site specific. More reliable data and information are therefore required for the development of mitigation and adaptation strategies to take full consideration of potential co-benefits and trade-offs. Individual agroecosystem models are typically well developed for the evaluation of certain goods and services, but not all. Integration of different models can help the assessment of internal consistency and provide more comprehensive analysis of multiple goods and services in a timelier manner [1].
• Along with monthly weather data, the tabulation of full timeseries, rather than multiple-year averages, exposes the inter-annual variations of various variables which are relevant for the quantification of goods and services. Apart from the average responses over time as reported in the corresponding published paper [1], these interlinked timeseries are essential for the examination of associated temporal variations for evaluation of resilience, correlations with weather conditions for responses to short term climate variations and for understanding their broader implications.
• Combined with similar work in other parts of the world with different natural environments, cropping varieties and fertilizer management, these site-specific data could also contribute to the better understanding of ecosystem services response to drastic changes in fertilizer management at broad scale.

## 2 Background

The increased use of synthetic nitrogen fertilizer in intensive modern farming systems has contributed to the current climate change crisis. It is a significant source of anthropogenic Greenhouse Gas (GHG) emissions; especially potent nitrous oxide (N_2_O). To achieve sustainable development and mitigate GHG emissions from the agricultural sector, it is crucial to understand the impacts of potential reductions in fertilizer use on various ecosystem goods and services, including crop production, GHG emissions and soil organic carbon (SOC) storage. While there are established individual agroecosystem models which can simulate some goods and services, rarely does any individual model simulate multiple goods and services. An alternative approach, applied extensively in climate science, but not currently in agroecosystem science, is to combine models in an ensemble. On this basis, models can be loosely integrated through the sharing of parameters and input data and exchange of intermediate and final outputs to ensure consistency in the data and information flow for modelling the complex atmosphere-plant-soil system with policy relevant scenarios related to fertilizer management. The idea was implemented and tested in an intensively instrumented research catchment in southwest England. The data introduced here have been used to derive annual averages of agroecosystem goods and services reported in a paper published in the Journal of Environment Management [1].

## 3 Data Description

Simulated data were split into 5 easily accessible workbooks with self-explanatory file names, including ‘Weather data’, ‘Yield and biomass data’, ‘N2O and CH4 data’, ‘CO2 data’ and ‘SOC data’. ‘Weather data’ described one of the key drivers affecting air-soil-plant interactions and the others covered modelled indicators for ecosystem services: provisioning, regulatory and supporting, respectively. For weather data, monthly total rainfall, average air temperatures and calculated potential evapotranspiration (PET) using the Penman–Monteith's equation [5] were provided for both baseline and future time periods. For modelled outputs, crop specific data were provided in separate worksheets (‘WW’ for winter wheat, ‘MZ’ for maize, ‘WB’ for winter barley, ‘SB’ for spring barley, ‘WB’ for winter barley, ‘WOSR’ for winter oil seed rape and ‘Grass’ for ‘improved grass’). Within each worksheet, results from different climate scenarios (baseline, RCP2.6, RCP4.5, RCP8.5) were tabulated in blocks from left to right and their cells are shaded in different colours for clarity. The results from different fertilizer regimes were arranged from top to bottom within each data block. Within each data block, results for different soil types (Denbigh, Hallsworth, Hlalstow and Neath soil series) [11] were shown in individual columns with appropriate units shown. Within each workbook, a shared ‘Metadata’ worksheet was attached which provides more information and external links where appropriate for the various factors considered for the construction of scenarios, such as climate conditions, soil types, crop types and their fertilizer application rates and regimes.

## 4 Experimental Design, Materials and Methods

The data provided is the result of a factorial based modelling exercise with two integrated process-based models: SPACSYS and RothC. The daily time step SPACSYS model was used to generate detailed crop development information which was then used for the parameterization of the monthly RothC model; e.g., estimated leaf-area index to determine the binary soil cover condition (covered vs not covered) and estimated annual biomass for the determination of fresh SOC inputs. The main objectives were to quantify the long-term (30 years) effects of the reduction of fertilizer application rates on multiple agroecosystem goods and services as indicated by crop yield, methane and nitrous oxide emissions, carbon dioxide release from soils and plants and SOC sequestration in the upper River Taw Observatory (URTO). More information about the case study catchment can be found here: https://www.rothamsted.ac.uk/project/upper-river-taw-observatory-urto.

Four levels of fertilizer application rates were considered: Business as Usual (BAU) rates, a 10 % reduction in BAU rates, 30 % reduction in BAU rates and 50 % reduction in BAU rates. The selection of these reduction percentages was aimed to cover a wide range of potential changes, considering the recent extant pressures from soaring fertilizer prices and volatile supplies [6]. The BAU rates were estimated from published 5-year (2017–2021) average rates from the British Survey of Fertiliser Practices [7]. For the various crops included in the study, data for the ‘Cereal’ robust farm type [8] was used. The rates for ‘Other livestock grazing’ [7] were adopted for grassland considering the dominance of lowland grazing in the study catchment. While no changes were made for grassland, application frequencies and timings were adjusted for the crops considered based on expert judgement (Table 1).

**Table 1:** Fertilizer application splits and timings.

	First	Second	Third
*BAU or 10 % reduction relative to BAU*			

Winter wheat	1 March (20 %)	15 April (80 %)	
Winter oilseed rape	19 August (50 %)	2 April (50 %)	
Winter barley	2 March (30 %)	15 April (70 %)	
Spring barley	19 February (40 %)	15 April (60 %)	
Maize	29 April		
Ryegrass	15 Apr (35 %)	15 Jun (35 %)	15 Jul (30 %)

*30 % reduction relative to BAU*			

Winter wheat	1 March (100 %)		
Winter oilseed rape	19 August (50 %)	2 April (50 %)	
Winter barley	2 March (100 %)		
Spring barley	19 February (40 %)	15 April (60 %)	
Maize	29 April (100 %)		
Ryegrass	15 Apr (35 %)	15 Jun (35 %)	15 Jul (30 %)

*50 % reduction relative to BAU*			

Winter wheat	1 March (100 %)		
Winter oilseed rape	19 August (50 %)	2 April (50 %)	
Winter barley	2 March (100 %)		
Spring barley	19 February (100 %)		
Maize	29 April (100 %)		
Ryegrass	15 Apr (35 %)	15 Jun (35 %)	15 Jul (30 %)

The main factors affecting the values of the modelled outcomes were considered, including climate conditions, soil types and crop types. Observed daily weather data at a local weather station (North Wyke) for the period 1985 to 2015 and projected daily weather under three different climate scenarios (RCP2.6, RCP4.5, RCP8.5) from the Inter-Sectoral Impact Model Intercomparison Project [9] for years spanning 2021 to 2050 were used to characterize the baseline and future climates, respectively. Crops with significant spatial coverage and high nitrogen demand were selected for the modelling exercise: winter wheat, spring barley, winter barley, winter oilseed rape, maize for forage and ryegrass as being generally representative of improved grassland in the study catchment. The last two were included because of their strong connections with dominant farming activities in the study area, i.e., lowland grazing. Four dominant soil series used for agriculture in the study catchment were included. These are Denbigh (series number 305), Hallsworth (series number 702), Halstow (series number 703), and the Neath series (series number 1303). More information on these soil types can be found on the designated web site for the data product used (https://www.landis.org.uk/soilsguide/). Only grass was grown in the Neath series, so no other crops were modelled for this specific soil type. In summary, combinations of 4 fertilizer rates x 4 climate conditions × 3 soil types × 5 crop types were examined. In addition, 4 fertilizer rates × 4 climate conditions were also considered for improved grassland.

All crops and grass were grown continuously throughout the modelling period; i.e., no crop rotations and their potential effects were considered explicitly. To account for the active processes in non-growing seasons, simulations were run throughout the year. While no genetic traits for each crop / grass were modified, typical local management practices were used to parameterize the individual crops and grass. Ploughing was simulated to take place on the 10th September for winter wheat and winter barley, 30th July for winter oilseed rape, 30th January for spring barley and 20th April for maize, all with a depth of 20 cm. Sowing occurred between 2 or 3 weeks later. All simulations start on the dates as shown in the tabulated data. Full datasets were made available, but it is advised that the first two years should treated as ‘burn-in’ years. Other specific parameter settings can be obtained by contacting the first author.

Limited calibrations were undertaken under BAU conditions to ensure that the model setup for different crops generated satisfactory yield predictions as indicated by the published data for multiple-years for the southwest region of England (https://www.gov.uk/government/statistics/cereal-and-oilseed-rape-production). These comparisons were summarised in the associated publication [1]. Parameterization of soil profile and soil layer specific properties were based on the attribute tables in NatMap [10,11], including ‘HORIZONHydraulics.csv’ and ‘HORIZONFundamental.csv’. Simple linear interpolation was employed to derive the initial soil status for setting up different soil layers for the modelling. Nitrogen related nutrient contents, expressed in g N m^−2^, are shown in Table 2 for the soil types under examination.

**Table 2:** Nitrogen related nutrient contents (g N m^−2^).

Denbigh				
Soil layer	Nitrate	Ammonium	Organic nitrogen	
(m)			Arable	Grass
0–0.1	1.20	0.82	388	452
0.1–0.2	1.20	0.82	388	452
0.2–0.3	1.20	0.82	291	242
0.3–0.4	0.50	0.20	116	212
0.4–0.6	0.04	0.04	209	355
0.6–0.8	0.00	0.00	161	211
0.8–1.0	0.00	0.00	84	84
1.0–1.35	0.00	0.00	50	50

**Hallsworth**				

Soil layer	Nitrate	Ammonium	Organic nitrogen	
(m)			Arable	Grass

0–0.1	1.20	0.82	410	477
0.1–0.2	1.20	0.82	410	370
0.2–0.3	1.20	0.82	157	108
0.3–0.4	0.50	0.20	121	108
0.4–0.6	0.04	0.04	209	196
0.6–0.8	0.00	0.00	173	173
0.8–1.0	0.00	0.00	147	147
1.0–1.35	0.00	0.00	257	257

**Halstow**				

Soil layer	Nitrate	Ammonium	Organic nitrogen	
(m)			Arable	Grass

0–0.1	1.20	0.82	306	364
0.1–0.2	1.20	0.82	306	364
0.2–0.3	1.20	0.82	99	132
0.3–0.4	0.50	0.20	71	96
0.4–0.5	0.04	0.04	71	96
0.5–0.6	0.00	0.00	58	58
0.6–0.75	0.00	0.00	87	87

**Neath**				

Soil layer	Nitrate	Ammonium	Organic nitrogen	
(m)			Grass	

0–0.1	1.20	0.82	466	
0.1–0.2	1.20	0.82	466	
0.2–0.3	1.20	0.82	368	
0.3–0.4	0.50	0.20	140	
0.4–0.5	0.04	0.04	140	
0.5–0.6	0.00	0.00	55	
0.6–0.7	0.00	0.00	83	
0.7–0.9	0.00	0.00	0	

Both agroecosystem models are freely available for scientific research. SPACSYS can be download from the following link: https://www.rothamsted.ac.uk/rothamsted-spacsys-model and RothC from the following link: https://www.rothamsted.ac.uk/rothamsted-carbon-model-rothc. Some supportive documentation for each model is provided on these links. The Excel version of RothC is available upon request to Asma Jebari (asma.jebari@rothamsted.ac.uk). Some key routines used for the data extraction from the SQL server can be found in the supplementary file.

## Limitations

The simulated data were not designed to be a climate impact assessment with full uncertainty analysis. Only a single realisation of future climate scenarios was considered. The data presented does not represent the feasible full ranges of ecosystem service responses to fertilisation. The results are also only applicable to areas with similar baseline weather conditions and projected future climates. For some components of the ecosystem services considered herein, e.g., CO_2_ emissions in conjunction with soil carbon mineralisation, either of the two models could be used for quantification. For the current study, no thorough comparisons have been undertaken to assess the compatibility of the outputs from the two individual models. Instead, expert judgement was used to select the outputs from one model.

## Ethics Statement

Hereby, we (Yusheng Zhang, Lianhai Wu, Asma Jebari, Adrian L. Collins) have declared that we have read and follow the ethical requirements for publication in Data in Brief and confirm that the current work does not involve human subjects, animal experiments, or any data collected from social media platforms.

## CRediT authorship contribution statement

**Yusheng Zhang:** Conceptualization, Formal analysis, Investigation, Methodology, Software, Validation, Visualization, Writing – original draft, Writing – review & editing. **Lianhai Wu:** Conceptualization, Data curation, Investigation, Validation, Software, Writing – original draft. **Asma Jebari:** Data curation, Investigation, Validation, Software, Writing – original draft. **Adrian L. Collins:** Conceptualization, Funding acquisition, Project administration, Resources, Supervision, Writing – original draft, Writing – review & editing.

## Data Availability

Simulation dataset of annual yields, GHG emissions and SOC stocks under current and projected climate conditions for major crops with current and reduced fertiliser rates in Southwest, England (Original data) (Zenodo)

## Appendix Supplementary materials

Image, application 1
